# Right Ventricular Outflow Tract Tachycardia with Structural Abnormalities of the Right Ventricle and Left Ventricular Diverticulum

**DOI:** 10.1155/2015/708687

**Published:** 2015-10-05

**Authors:** Bortolo Martini, Nicola Trevisi, Nicolò Martini, Li Zhang

**Affiliations:** ^1^Cardiovascular Unit, Alto Vicentino Hospital, Via Gioberti 9, 36016 Thiene, Italy; ^2^San Raffaele Hospital, 20132 Milan, Italy; ^3^Medical College, Ferrara Medical University, 44121 Ferrara, Italy; ^4^Lankenau Medical Center, Wynnewood, PA 19096, USA; ^5^Lankenau Institute for Medical Research, Wynnewood, PA 19096, USA; ^6^Jefferson Medical College, Thomas Jefferson University, Philadelphia, PA 19107, USA

## Abstract

A 43-year-old woman presented to the emergency room with a sustained ventricular tachycardia (VT). ECG showed a QRS in left bundle branch block morphology with inferior axis. Echocardiography, ventricular angiography, and cardiac magnetic resonance imaging (CMRI) revealed a normal right ventricle and a left ventricular diverticulum. Electrophysiology studies with epicardial voltage mapping identified a large fibrotic area in the inferolateral layer of the right ventricular wall and a small area of fibrotic tissue at the anterior right ventricular outflow tract. VT ablation was successfully performed with combined epicardial and endocardial approaches.

## 1. Introduction

Right ventricular outflow tract tachycardia (RVOT-VT), while being often classified as an idiopathic ventricular arrhythmia, has been found to have structural cardiac abnormalities in some cases [1]. Such cases probably represent an atypical manifestation of arrhythmogenic right ventricular dysplasia cardiomyopathy (ARVD/C). Left ventricular diverticulum (LVD), a rare cardiac malformation, can be a standalone condition or associated with other cardiac abnormalities. However, LVD itself rarely causes ventricular arrhythmias. Here we report a case with a rare combination of atypical ARVC/D with an LVD and with RVOT-VT as a primary presentation.

## 2. Case Report

**Figure 1:**
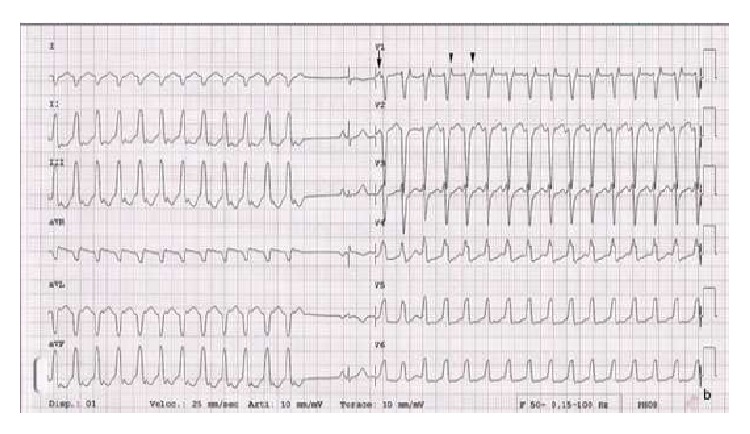
VT in LBBB morphology and inferior QRS axis. The first arrow points to a dissociated P wave in V1; then retrograde conducted 1 : 1 P waves follow (arrowheads).

**Figure 2:**
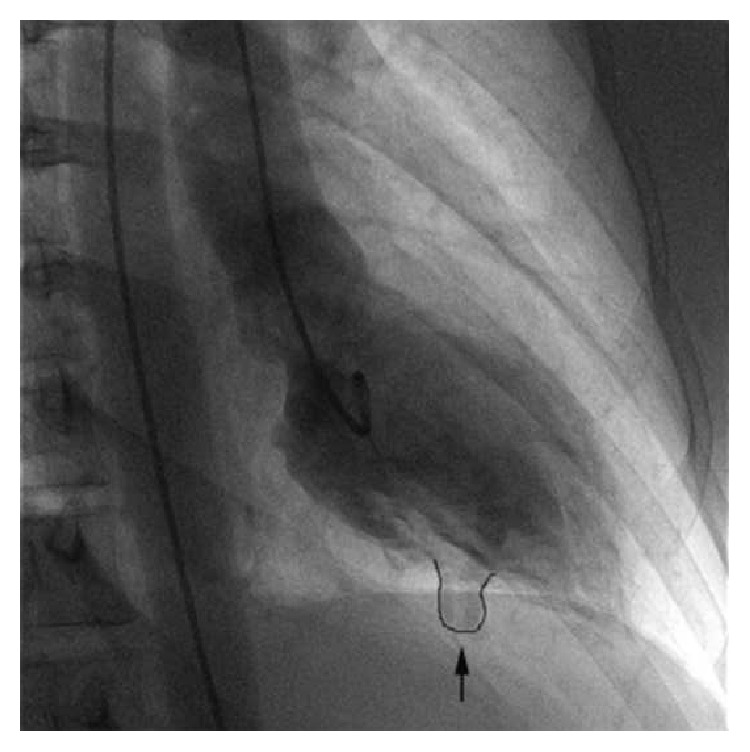
Left ventriculogram showing a left ventricular diverticulum (arrow).

**Figure 3:**
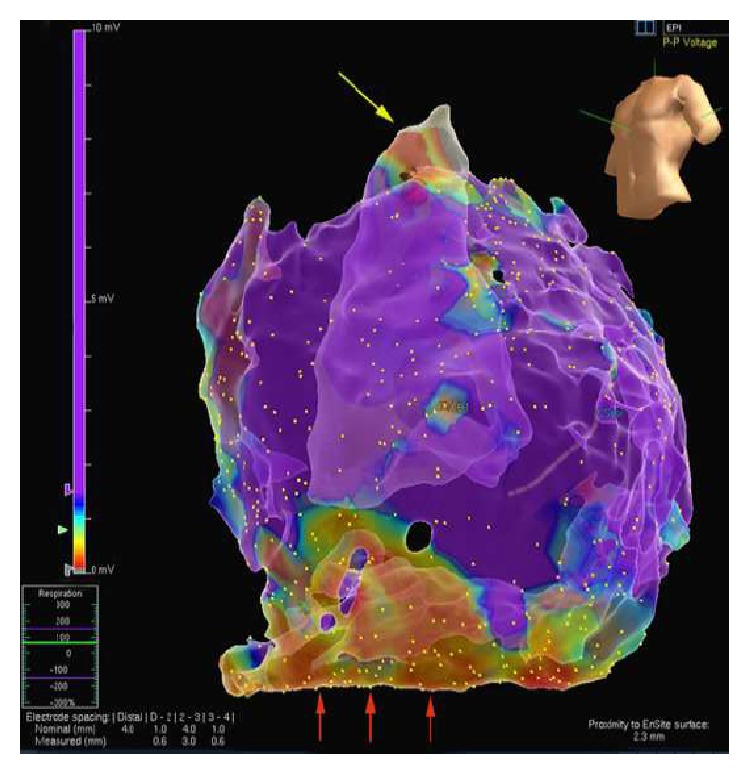
Epicardial electroanatomic mapping of the right ventricle. Bipolar mapping of the epicardial surface shows a small area of fibrotic tissue in the distal RVOT (yellow arrow) and a larger area of fibrotic tissue involving inferolateral free wall of the right ventricle (red arrows).

A 43-year-old woman presented to the emergency room (ER) with palpitations, dizziness, and shortness of breath. The patient did not have chest pain or syncope. She had a 2-year history of palpitations. An ECG and an echocardiogram performed six years prior to her presentation were normal accordingly. She denied family history of premature sudden death and coronary artery disease. An ECG done in the ER showed a sustained ventricular tachycardia (VT) with a rate of 218 bpm. The QRS pattern was in left bundle branch block (LBBB) morphology with inferior axis (Figure 1). Retrograde 1 : 1 P waves were present. Adenosine temporarily interrupted the tachycardia. However, multiple runs of nonsustained VT, all in LBBB morphology, were recorded on telemetry. After intravenous administration of amiodarone all ventricular arrhythmias were abolished. The ECG while on amiodarone showed sinus rhythm, low QRS amplitudes in limb leads, T wave inversion in V1–V3, and QTc prolongation. In the following days the inverted T waves in the right precordial leads gradually became upright after the discontinuation of amiodarone, but VTs reappeared. Flecainide failed to abolish VT. The patient was finally started on atenolol with good results. Cardiac angiography revealed patent coronary arteries, normal right ventricle, and a left ventricular diverticulum (LVD) with a distinct morphology (Figure 2). LVD was also confirmed by cardiac magnetic resonance imaging (CMRI). Signal averaged ECG study was negative. An electrophysiology study was performed which showed a normal endocardial voltage mapping. However, the epicardial mapping divulged a small area of fibrotic tissue at the anterior RVOT and a large area of fibrotic tissue in the inferolateral portion of the RV (Figure 3). Activation mapping demonstrated that the earliest tachycardia exit point was in the endocardial RVOT, while the best pace-mapping was in the facing epicardial surface. VT ablation was successfully performed with combined epicardial and endocardial approaches. After the ablation, this patient has been event-free for the last ten months without any drug therapy. Since there is a large area of fibrotic tissue formation on the epicardial surface of the right ventricle, long-term follow-up was advised.

## 3. Discussion

The occurrence of VT in LBBB morphology along with a large area of fibrotic tissue in the RV epicardium supports the diagnosis of a localized form of arrhythmogenic right ventricular dysplasia/cardiomyopathy (ARVD/C) even though these findings do not fulfil the current diagnostic criteria for ARVD/C [2]. Inverted T waves in leads V1–3 were present only during amiodarone infusion. The antiarrhythmic effect of amiodarone is mainly by delaying the Phase III ventricular repolarization which can result in T wave abnormalities. Furthermore, the echocardiogram and CMRI were all negative for the RV assessment. Without the epicardial mapping, this patient could have been mistakenly thought to have an idiopathic RVOT-VT.

Despite different opinions given many years ago [3, 4], RVOT-VT has long been retained as the typical idiopathic ventricular arrhythmias. We view some of those so-called RVOT-VTs that are actually the minor forms of ARVD/C [5] since the affected area in the RV is limited or in the regions outside of the “Dysplasia Triangle” [5]. The atypical forms of ARVD/C are mostly underdiagnosed due to the lack of typical presentations on ECG, angiography, CMRI, and other aspects. In ARVD/C the most frequently involved areas of the RV wall are the posterior base, apex, and the RVOT. In the latter, a marked RVOT dilatation can be seen on CMRI [6]. However, if the fibrofatty substitution is limited to a small area or epicardium of the RV, only endocardial and/or epicardial mapping can detect these abnormalities [7].

In our case only epicardial voltage mapping revealed the key structural cardiac abnormalities: a small fibrotic area in the anterior epicardial RVOT and a large area of fibrotic tissue formation in the inferolateral epicardial layer of the RV (Figure 3). These findings could arguably support the diagnosis of localized form of ARVD/C [2, 8, 9].

On the other hand, the ventriculogram and CMRI of our patient clearly support the diagnosis of an isolated diverticulum (Figure 2). Cardiac diverticulum is a rare cardiac malformation that can affect either ventricles, but LVD is the most common [10]. Recently Ohlow et al. provided a comprehensive review of 809 cases retrieved from the literature since 1816 [10]. Accordingly, most patients with LVD experience a benign clinical course. Nonetheless, those with severe forms of LVD ventricular wall rupture can occur [10]. Ventricular arrhythmia is uncommon. In those with documented VT, the ectopics are in LV origin therefore showing a RBBB pattern [11]. In contrast VT in our case has a LBBB morphology, indicating that the origin is in the RV (Figure 1). Left ventricular aneurysms can develop in ARVD/C; thus it should be differentiated from a cardiac diverticulum. Several important diagnostic characteristics can help distinguish these two entities. A cardiac diverticulum, as in our patient, usually has a narrow neck and a systolic flow pattern from the diverticulum to the ventricle and is usually small in size and has a circular shape [12].

## 4. Conclusions

LVD can be a standalone condition or associated with other congenital heart diseases. To our knowledge this is the first case of LV diverticulum coincidently coined with an atypical form of ARVD/C. This case could have been easily misdiagnosed as an idiopathic RVOT-VT otherwise. Identifying the underlying structural abnormality in patients with VT is not an easy task sometimes. Achieving the correct diagnosis is not simply based on detecting common criteria but may require various diagnostic modalities such as clinical and ECG analysis, echocardiography, angiography, tissue diagnosis, CMRI, genetic studies, and electroanatomic mapping using both the epicardial and endocardial approaches. Question remains as to whether ARVD/C and LVD in this case are “incidentaloma” or related to each other embryologically.

## Key Teaching Points

1. With the emerging new technology, some patients with right ventricular outflow tract tachycardia (RVOT-VT) are found to have structural cardiac abnormalities. Fibrotic tissue formation in the right ventricle is likely the cause, and it sometimes can only be detected by epicardial mapping. These patients have been considered as having an atypical form of arrhythmogenic right ventricular dysplasia cardiomyopathy (ARVD/C).
2. Left ventricular diverticulum (LVD) itself rarely causes arrhythmias. When the imaging finding cannot explain the symptom, further investigation is necessary.
3. A combination of ARVD/C and LVD is extremely rare. Whether it is an incidental finding “incidentaloma” or these two entities are related to each other embryologically or pathologically will warrant further investigation.
